# A *Wolbachia* coinfection in the common bed bug

**DOI:** 10.1093/ismeco/ycag197

**Published:** 2026-07-08

**Authors:** Hillary E Davis, Joseph Torres, Meaghan J Adler, Benjamin J Parker

**Affiliations:** Department of Emergency Medicine, University of North Carolina at Chapel Hill, Chapel Hill, NC, 27599, United States; Wake Forest Emergency Providers, Alamance Regional Medical Center, Burlington, NC, 27215, United States; Department of Biology, University of North Carolina at Chapel Hill, Chapel Hill, NC, 27599, United States; Department of Biology, University of North Carolina at Chapel Hill, Chapel Hill, NC, 27599, United States; Department of Biology, University of North Carolina at Chapel Hill, Chapel Hill, NC, 27599, United States

**Keywords:** *Wolbachia*, bed bugs, symbiosis, horizontal transmission, biocontrol

## Abstract

The common bed bug (*Cimex lectularius*) relies on an obligate mutualism with the *Wolbachia* strain *w*Cle to supplement B vitamins deficient in human blood. Using metatranscriptomic and metagenomic sequencing of hospital-collected bed bugs, we found that some individuals also harbor a second strain of *Wolbachia* (*w*Chem). Using publicly available data we showed that *w*Chem is distributed in bed bugs worldwide at intermediate frequencies and may have moved recently between *C. lectularius* and *Cimex hemipterus*, the tropical bed bug, which also feeds on human hosts. We found that *w*Chem encodes a highly expressed *cifA/B* operon in males and females, consistent with cytoplasmic incompatibility, a reproductive manipulation strategy used by *Wolbachia* to increase in frequency in host populations. Together, these results demonstrate that some bed bugs harbor a *Wolbachia* coinfection of a nutritional mutualist and a potentially manipulative facultative symbiont. This discovery identifies a previously hidden aspect of bed bug biology with significant implications for its evolution, spread, and potential control.

The common bed bug (*Cimex lectularius*) has resurged as a public health issue, driven by increased globalization and insecticide resistance [1]. Bed bugs harbor an obligate *Wolbachia* endosymbiont (*w*Cle) that supplements B-vitamins needed with blood feeding [2, 3]. This mutualism contrasts with *Wolbachia* strains that spread via reproductive manipulation, which are traits exploitable for pest biocontrol [4, 5]. Searches for additional heritable microbes have uncovered facultative microbial symbionts in bed bugs [6], but none have been linked to reproductive manipulation.

Using metagenome and metatranscriptome sequencing of samples from a hospital emergency room, we found that some bed bugs harbor a coinfection of two distinct *Wolbachia* strains. Four out of 15 samples (26.7%) had reads that mapped to *Wolbachia* strain *w*Chem PL13 (*Candidatus* Wolbachia massiliensis GCA_014771645 [7]; hereafter *w*Chem) in addition to *w*Cle (Fig. 1A). In whole-body RNAseq libraries, *w*Chem reads averaged 46.8% of total *Wolbachia* reads (range 30.2–71.4%), suggesting substantial relative abundance. From nanopore sequencing of hemolymph gDNA (see the Supplementary S1), we assembled a complete *w*Cle genome (1.294 Mbp; BUSCO 95.1% C; 2.9% D; 0.9% F; 1.2% M) and a partial *w*Chem genome (0.557 Mbp; BUSCO 38.5% S; 0.0% D; 2.6% F; 58.8% M) from a single individual. Our *w*Cle assembly reflected 78.6× coverage, compared to 5.5× coverage for *w*Chem and 9.5× coverage for the bed bug host. The low coverage we obtained for *w*Chem likely reflects our use of hemolymph for metagenome sequencing rather than whole-body DNA extraction. We found no evidence that *w*Chem reads originated from a *Wolbachia* genome integration, as has been observed in other systems [8, 9]. BLAST screening detected no chimeric reads spanning bed bug-*w*Chem boundaries, and *w*Chem transcripts mapped to regions absent from our partial genome assembly, indicating that limited sequencing coverage, rather than genome integration, explains the incomplete assembly.

**Figure 1:**
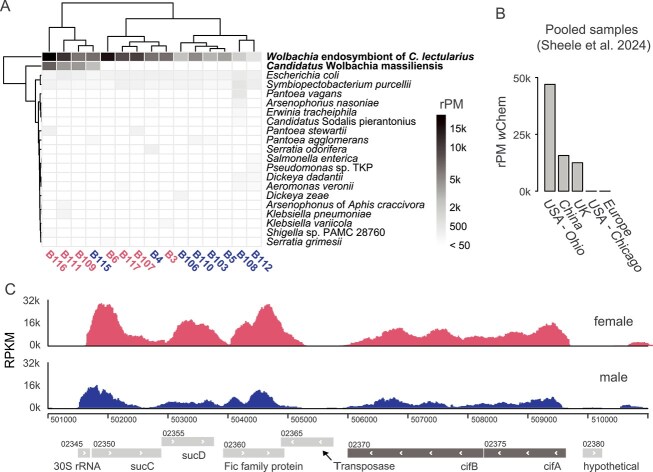
A. Reads per million (rPM) mapped from bed bug metatranscriptome sequencing by bacterial species. The 15 sequenced samples are shown along the bottom of the figure, colored by male (blue) or female (red). Bacterial species ID of the best BLAST hit of assembled contigs are shown along the right of the figure, with the two *Wolbachia* strains at the top in bold. The heatmap shows rPM as indicated in the key, and dendrograms are shown grouping samples and microbial taxa at the top and left of the figure. B. rPM of reads mapped to *w*Chem from data collected by Sheele *et al.* 2024. Each sample is shown along the bottom of the figure. C. Expression RPKM values across 10 kb of the *w*Chem genome from a male and a female RNAseq sample. Gene annotations are shown at the bottom of the figure with the *cif* operon in dark grey.

*Wolbachia* strain *w*Chem is a recently described “supergroup T” lineage, originally isolated from the tropical bedbug *Cimex hemipterus* and grown in cell culture in order to produce a complete genome sequence (GCA_014771645) [7]. We found that *w*Chem genomes from *C. hemipterus* and *C. lectularius* are nearly identical, sharing 99.4% average nucleotide identity (ANI-B) and 99.2% nucleotide identity across BUSCO genes. In contrast, the obligate *w*Cle strains (*C. lectularius:* GCA_000829315; *C. hemipterus:* GCA_003704325) from these same host species exhibit significantly higher divergence (89.4% ANI-B) [4]. These results suggest that *w*Chem diverged more recently than the obligate *w*Cle strains, supporting a model of recent horizontal transmission between *Cimex* species. Within *C. lectularius, w*Cle and *w*Chem share 86.7% ANI-B and 88.4% identity across BUSCO genes. To assess *w*Chem’s global distribution, we screened a published *C. lectularius* metatranscriptomic dataset (from a study that was primarily focused on viral diversity) [10], and we detected *w*Chem reads in populations from the USA, UK, and China (Fig. 1B). We also found high numbers of reads with homology to *w*Chem in 3/3 *C. hemipterus* metatranscriptome samples on NCBI (PRJNA1081303) [5], but more sampling is needed to assess the frequency of *w*Chem in tropical bed bugs. Together, these results indicate that *w*Chem occurs across multiple continents, is found in some but not all *C. lectularius* individuals, and potentially underwent horizontal transmission between *Cimex* species.

*w*Chem was presumed to function as a nutritional symbiont in the original publication describing this microbe in *C. hemipterus* [7]. In other bed bug species, subsequent work has found a pattern of frequent symbiont acquisitions and replacement of the primary nutritional *Wolbachia* [4]. However, although the *w*Chem genome encodes several B-vitamin pathways, the published reference genome for *w*Chem from *C. hemipterus* does not encode for the horizontally acquired biotin operon [7] that is key to the nutritional mutualism [2, 3, 7], and it was not investigated in the original paper whether the *C. hemipterus* insects from which the cultured *w*Chem was isolated also harbored *w*Cle. It is therefore currently unclear if *w*Chem functions as a nutritional symbiont in *Cimex* hosts, and further studies are needed.

Cytoplasmic incompatibility (CI) is one of the most widespread symbiont reproductive manipulations, mediated by paired *cifA* and *cifB* effectors [11, 12]. These proteins modify sperm during spermatogenesis and induce embryonic lethality when infected males mate with females lacking a compatible infection, thereby biasing reproduction in favor of infected lineages. We found strong expression of a *cifA* homolog (ID128_RS02375) in *w*Chem harbored by both male and female samples (Fig. 1C). This gene is immediately upstream of a 754aa uncharacterized protein (ID128_RS02370) that is also highly expressed in males and females (Fig. 1C). BLASTP results of the protein sequence against the RefSeq database identified multiple homologs with full-length cover including hits annotated as *cifB*. Comparison of the protein sequence against the AlphaFold Database clustered at 50% sequence identity (AFDB50) identified a top hit to a protein from the *Wolbachia* endosymbiont of *Cylisticus convexus* (*w*Cc), a strain that induces unidirectional CI in its host [13], annotated as a PD-(D/E)XK nuclease (A0A369RET0; 57% sequence identity across residues 1–732; e-value 2.75 × 10^−98^), and the predicted model exhibited high confidence (average pLDDT = 82.75). Together with its genomic proximity to *cifA* and strong expression, this homology supports the interpretation that ID128_RS02370 is a plausible *w*Chem *cifB* homolog. However, though *cifB*-transcript levels have been shown to largely explain CI across strains of *Wolbachia* [14], small mutations can disrupt or weaken CI [15] and experimental tests and molecular characterization of this gene are needed. Of note, studies of crossing success between human- and bat-associated lineages of *C. lectularius* have produced conflicting results [16, 17], and future studies of reproductive barriers in bed bugs should take *w*Chem infection into account.

*Wolbachia* coinfections typically involve two reproductively manipulative strains coinhabiting reproductive tissues [18], but *w*Cle occupies specialized bacteriomes in addition to ovaries. How two heritable symbionts with different functions are segregated among host tissues remains an open question. High expression of the *cif* operon suggests *w*Chem may spread via CI, though its intermediate population frequency suggests weak CI. The high nucleotide identity between *w*Chem in *C. lectularius* and *C. hemipterus* is consistent with recent horizontal transmission between species that have come into contact through globalization [19], and low frequency in some populations may reflect recent introduction. Future work should address *w*Chem's transmission dynamics, tissue tropism, and potential utility for biocontrol in a context of rising pesticide resistance.

## Supplementary Material

Supplemental_Info_resub2_ycag197

## Data Availability

Raw nanopore reads from metagenome sequencing, along with genomes for *w*Cle and *w*Chem (partial), were deposited to NCBI under Bioproject PRJNA1425112. Raw RNAseq reads from metatranscriptome sequencing was deposited to NCBI under Bioproject PRJNA1428758.
