# Supplementary material for: A Wolbachia coinfection in the common bed bug
Source: ISME Commun. 2026 Jul 8;6(1):ycag197. doi: 10.1093/ismeco/ycag197 (PMC13455600; doi:10.1093/ismeco/ycag197)
Supplement: Supplementary file 1 [file supplemental_info_resub2_ycag197.pdf]

SUPPLEMENTAL INFORMATION

Bed bug collection: We collected 15 adult bedbugs for RNAseq from the University of Tennessee Medical Center's Emergency Department over several months in 2024. Samples were stored in sterile plastic collection jars during transport, and were then moved to -80°C. We collected the single bed bug used for nanopore gDNA sequencing from Alamance Regional Center Emergency Department in Burlington, North Carolina, USA in 2025.

RNA extraction, RNAseq library prep, and sequencing: We extracted RNA from 15 bed bug samples individually using a Trizol/BCP extraction. Each insect was crushed using a sterile RNAse-free pestle in 800μL Trizol and incubated for 5m at room temperature. We then added 107μL of 1-Bromo-3-chloropropane (Sigma Aldrich) for a 1:7.5 ratio of trizol/BCP based on pilot extractions. We mixed samples by hand for 20s and incubated for a further 5m at room temperature. We centrifuged samples at 4°C at 12,000G for 15m and removed the aqueous layer, to which we added 400μL isopropanol, inverted the tubes for 20s, and incubated at room temperature for 10m. We then spun samples at 4°C for 10m at 12,000G to form a pellet, which we then isolated and washed using 75% EtOH. We air-dried the pellet at room temperature for 10m, and then resuspended in RNAse-free water. We treated the samples with DNAse I to remove genomic DNA and used the Zymo Clean and Concentrator kit to purify samples for RNAseq. Sequencing was conducted at Novogene, Inc. using a meta-transcriptome library prep. Library prep included prokaryotic and eukaryotic rRNA depletion with the Illumina Ribo-Zero depletion kit, and 150bp paired-end sequencing was carried out on an Illumina NovaSeq X Plus instrument to at least 9 billion bp per sample. Adapter trimming was performed by Novogene as part of standard data delivery.

Metagenome sequencing of genomic DNA via nanopore: We extracted high molecular weight (HMW) genomic DNA from bed bug hemolymph using a modified anion-exchange protocol adapted for Nanopore sequencing. We collected hemolymph from an adult bedbug by stabbing the insect in the thorax with a glass needle and then centrifuging the carcass through a 10 micron filter at 20,000G at 4°C directly into Zymo DNA/RNA Shield. We lysed samples in a custom buffer (Guanidine-HCl, Triton X-100, NaCl) supplemented with RNase A (37°C, 30m) followed by Proteinase K digestion (50°C, 3h). After clarifying the lysate via centrifugation, we purified DNA using a Qiagen Genomic-tip 20/G, which involved washing the column with buffer and eluting DNA in pre-warmed (50°C) Buffer QF. We precipitated the elute overnight at room temperature using isopropanol and glycogen, pelleted at 4°C, and then washed each pellet twice with cold 80% ethanol. We resuspended the resulting HMW DNA in a low-EDTA buffer (10 mM Tris-HCl, pH 8.5, 0.1mM EDTA) and solubilized at 4°C for 48 hours. We used ligation sequencing kit (SQK-LSR114) with V14 chemistry and a 10.4.1 flow cell following the manufacturer's recommended protocols with two slight modifications. We used 100μL of AMPure XP beads rather than the recommended volume, and we incubated the beads for 20m on a hula mixer with gentle rotation. We washed the final library with Long Fragment Buffer and ran the flow cell for 24h.

RNA sequencing results & sample sex identification. We generated an average of 12.9G base pairs of sequence data per sample, corresponding to an average of 86,229,302 paired-end reads. We used czid.org [1] for an initial survey of the species origin of reads in metatranscriptome samples, using the Illumina Metatranscriptome pipeline under default settings with of removal of human and bed bug host reads, filtering to >50 rPM per sample. We repeated this analysis (again using the CZID metagenome pipeline on default settings) using reads from NCBI accessed through BioProject IDs PRJNA972449 for *C. lectularius* and PRJNA1081303 for *C. hemipterus*.

We then aligned the raw reads to a combined reference file that included human (GRCh38.p14), bedbug (Clec_2.1), and *Wolbachia* genomes using STAR (STAR --runMode genomeGenerate --runThreadN [threads] --genomeDir [output directory] --genomeFastaFiles [combined reference FASTA] --sjdbGTFfile [combined reference GTF] --sjdbOverhang 59 --genomeSAindexNbases 9; STAR --runThreadN [threads] --genomeDir [output directory] --readFilesIn [R1 fastq] [R2 fastq] --outFileNamePrefix [output prefix]). Gene-level read counts were generated using htseq-count (htseq-count --format=sam --order=pos --stranded=no --type=gene --idattr=ID --nonunique all [input SAM file] [combined reference GFF] > [output counts file]). We used these reads to confirm our visual identification of adult male and female bed bugs by manually inspecting read counts for four vitellogenin-like genes in RNAseq libraries (LOC106673815, LOC106673813, LOC106667186, and LOC106663463), verifying high expression of these genes in females and low/no expression in males.

***w*Chem genome assembly**: We assembled nanopore metagenomic reads using canu v2.3 with the genome size parameter set to 50 Mb to retain contigs from multiple organisms present in the metagenome (canu -p bed-bug -d [output directory] genomeSize=50m utgReAlign=true corOverlapper=mhap usegrid=false -nanopore [input reads]). Resulting contigs were subjected to taxonomic assignment using nucleotide similarity searches with *BLAST+* (BLASTn) against a locally installed NCBI nt database using default parameters (blastn -db [nt database] -query [assembly contigs] -outfmt "6 qseqid staxids bitscore std stitle" -max_target_seqs 10 -max_hsps 3 -evalue 1e-20 -num_threads 60 -out [output file]). Contigs were assigned to the taxon of their top BLAST hit by bitscore. Mean coverage for each taxon was calculated by summing per-contig coverage values (reported by the assembler) for all contigs assigned to that taxon and dividing by the number of contigs. Contigs assigned to *Wolbachia* were extracted for downstream comparative analyses. We ran BUSCO v.5.8.3 using the rickettsiales_odb12 lineage to assess genome completeness (busco -m geno -i [input assembly] -l rickettsiales_odb12 -o [output directory]).

To screen for evidence of *w*Chem genome integration into the bed bug host, we used BLASTn to query metagenomic reads against the NCBI nt database (blastn -db [nt database] -query [metagenomic reads] -outfmt "6 qseqid staxids bitscore std stitle" -max_target_seqs 10 -out [output file]). We retained the top 5 hits per read and identified reads with hits to both *Candidatus* Wolbachia massiliensis (taxID: 1845000) and *Cimex lectularius* (taxID: 79782). Reads with hits to both taxa were considered putatively chimeric. Of 162 reads with hits to *w*Chem, none had hits to the bed bug genome. Further, *w*Chem transcripts mapped across the entire reference genome, with 97% of annotated genes (1,104/1,138) showing detectable expression.

We calculated average nucleotide identity by BLAST (ANI-B) to quantify genomic similarity among *Wolbachia* assemblies (pyani-plus anib --create-db --database [ANI database] fasta-dir/). We performed ANI-B comparisons between *w*Chem genomes assembled from *C. lectularius* and *C. hemipterus* (our assembly vs. GCA_014771645), between corresponding *w*Cle genomes from these host species (*C. lectularius:* GCA_000829315; *C. hemipterus:* GCA_003704325), and between *w*Chem and *w*Cle from within *C. lectularius* (our assembly vs. GCA_000829315). In addition to whole-genome ANI-B, we assessed nucleotide identity across conserved single-copy orthologs using BUSCO gene sets extracted from each assembly, and we calculated pairwise nucleotide identity across matched BUSCO loci using nucleotide alignments. To do this, we ran BUSCO v.5.8.3 on each genome using the rickettsiales_odb12 lineage (busco -i [input assembly] -l rickettsiales_odb12 -c 50 -m geno -o [output directory]). We collected single, fragmented, and duplicated BUSCO sequences from the output. We then mapped these sequences to each other pairwise using pyani-plus with the ANI-M method selected which carried out the pairwise alignments with NUCmer (pyani-plus anim --create-db --database [ANI database] fasta-busco-dir/). Matrices of aligned sequence lengths, similarity error counts,

average nucleotide identity (ANI) percentages, and minimum aligned percentage (of whole genome) were obtained directly from the default output files for each pairwise comparison.

Cif operon analysis: We searched for homologs of *cifA* using tBLASTn of GenBank via the NCBI Blast web interface: OK352257.1 against both *w*Chem genomes. We visualized the genomic region using IGV, and aligned .bam files from metatranscriptome data to these regions after normalizing by RPKM using deepTools bamCoverage v.3.5.6 (bamCoverage -b [input BAM file] -o [output file] --normalizeUsing RPKM --binSize 10 -p [threads]). A candidate *cifB* gene was initially identified through inspection of the genomic regions flanking *cifA,* followed by BLASTp against the nr database. Protein homology analyses were performed for the predicted 754-amino acid sequence encoded by a putative *cifB* gene by querying the sequence against the AlphaFold Protein Structure Database clustered at 50% sequence identity (AFDB v6; 50% sequence identity clusters; accessed February 2026).

1. Simmonds SE, Ly L, Beaulaurier J *et al.* Cz id: A cloud-based, no-code platform enabling advanced long read metagenomic analysis. *bioRxiv*. 2024 https://doi.org/10.1101/2024.02.29.579666
